# Supplementary material for: Resolving the full spectrum of human genome variation using Linked-Reads
Source: Genome Res. 2019 Apr;29(4):635–45. doi: 10.1101/gr.234443.118 (PMC6442396; doi:10.1101/gr.234443.118)
Supplement: Supplemental Material [file supp_gr.234443.118_Supplemental_longranger-2.2.zip › longranger-2.2.tar.bz2!/lr2.2-public/lib/stan/cmdstan/stan/doc/api/html/rev_2operators_2operator__subtraction_8hpp_source.html]

Stan: src/stan/agrad/rev/operators/operator\_subtraction.hpp Source File


|  |  |
| --- | --- |
|  | Stan  2.5.0  probability, sampling & optimization |


- Main Page
- Related Pages
- Namespaces
- Classes
- Files

- File List
- File Members

All Classes Namespaces Files Functions Variables Typedefs Enumerations Enumerator Friends Macros Pages

- src
- stan
- agrad
- rev
- operators

operator\_subtraction.hpp

Go to the documentation of this file.

1 #ifndef STAN\_\_AGRAD\_\_REV\_\_OPERATORS\_\_OPERATOR\_SUBTRACTION\_HPP

2 #define STAN\_\_AGRAD\_\_REV\_\_OPERATORS\_\_OPERATOR\_SUBTRACTION\_HPP

3

4 #include <stan/agrad/rev/var.hpp>

5 #include <stan/agrad/rev/internal/vv\_vari.hpp>

6 #include <stan/agrad/rev/internal/vd\_vari.hpp>

7 #include <stan/agrad/rev/internal/dv\_vari.hpp>

8 #include <boost/math/special\_functions/fpclassify.hpp>

9

10 namespace stan {

11  namespace agrad {

12

13  namespace {

14  class subtract\_vv\_vari : public op\_vv\_vari {

15  public:

16  subtract\_vv\_vari(vari\* avi, vari\* bvi) :

17  op\_vv\_vari(avi->val\_ - bvi->val\_, avi, bvi) {

18  }

19  void chain() {

20  if (unlikely(boost::math::isnan(avi\_->val\_)

21  || boost::math::isnan(bvi\_->val\_))) {

22  avi\_->adj\_ = std::numeric\_limits<double>::quiet\_NaN();

23  bvi\_->adj\_ = std::numeric\_limits<double>::quiet\_NaN();

24  } else {

25  avi\_->adj\_ += adj\_;

26  bvi\_->adj\_ -= adj\_;

27  }

28  }

29  };

30

31  class subtract\_vd\_vari : public op\_vd\_vari {

32  public:

33  subtract\_vd\_vari(vari\* avi, double b) :

34  op\_vd\_vari(avi->val\_ - b, avi, b) {

35  }

36  void chain() {

37  if (unlikely(boost::math::isnan(avi\_->val\_)

38  || boost::math::isnan(bd\_)))

39  avi\_->adj\_ = std::numeric\_limits<double>::quiet\_NaN();

40  else

41  avi\_->adj\_ += adj\_;

42  }

43  };

44

45  class subtract\_dv\_vari : public op\_dv\_vari {

46  public:

47  subtract\_dv\_vari(double a, vari\* bvi) :

48  op\_dv\_vari(a - bvi->val\_, a, bvi) {

49  }

50  void chain() {

51  if (unlikely(boost::math::isnan(ad\_)

52  || boost::math::isnan(bvi\_->val\_)))

53  bvi\_->adj\_ = std::numeric\_limits<double>::quiet\_NaN();

54  else

55  bvi\_->adj\_ -= adj\_;

56  }

57  };

58  }

59

98  inline var operator-(const var& a, const var& b) {

99  return var(new subtract\_vv\_vari(a.vi\_,b.vi\_));

100  }

101

113  inline var operator-(const var& a, const double b) {

114  if (b == 0.0)

115  return a;

116  return var(new subtract\_vd\_vari(a.vi\_,b));

117  }

118

130  inline var operator-(const double a, const var& b) {

131  return var(new subtract\_dv\_vari(a,b.vi\_));

132  }

133

134  }

135 }

136 #endif

boost::math::isnan

bool isnan(const stan::agrad::var &v)

Checks if the given number is NaN.

**Definition:** boost\_fpclassify.hpp:69

dv\_vari.hpp

stan::agrad::operator-

fvar< T > operator-(const fvar< T > &x1, const fvar< T > &x2)

**Definition:** operator\_subtraction.hpp:14

vd\_vari.hpp

stan::agrad::var::vi\_

vari \* vi\_

Pointer to the implementation of this variable.

**Definition:** var.hpp:40

unlikely

#define unlikely(x)

**Definition:** likely.hpp:9

var.hpp

stan::agrad::var

Independent (input) and dependent (output) variables for gradients.

**Definition:** var.hpp:27

vv\_vari.hpp


---

[ Stan Home Page ]

*© 2011–2014,
Stan Development Team.*
